# Supplementary material for: The Impact of Surgical Practice on Oncological Outcomes in Robot-Assisted Radical Hysterectomy for Early-Stage Cervical Cancer, Spanish National Registry
Source: Cancers (Basel). 2022 Jan 29;14(3):698. doi: 10.3390/cancers14030698 (PMC8833333; doi:10.3390/cancers14030698)
Supplement: Supplementary file 1 [file cancers-14-00698-s001.zip › cancers-1539366-supplementary.pdf]

SUPPLEMENTARY MATERIAL

Figure S1. Disease-free survival rates between surgical groups.

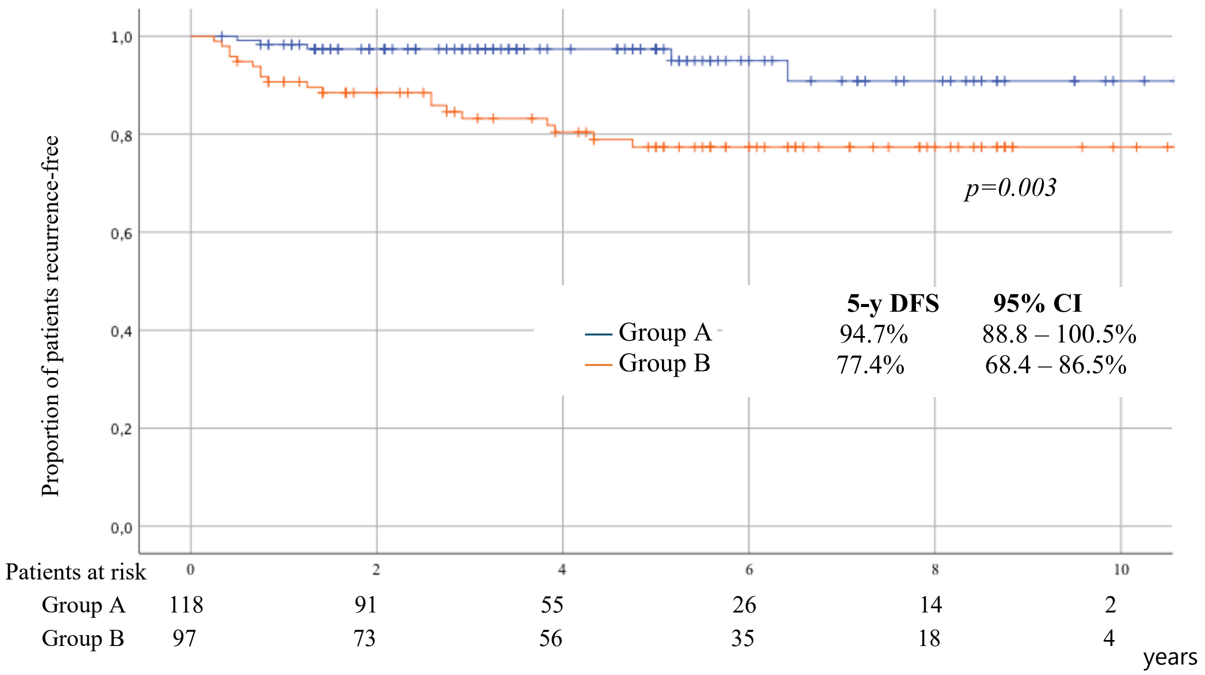

Graphic by Kaplan-Meier

Table S1. Complications of surgery

	Group A n=118	Group B n=97	p-value
Intraoperative, grade II-IV* (%)			
No	117 (99.2)	91 (93.8)	0.028
Urinary	1 (0.8)	5 (5.2)	
Intestinal	0	1 (1)	
Postoperative, grade II-IV* (%)			
No	112 (94.9)	85 (87.6)	0.055
Urinary	1 (0.9)	4 (4.1)	
Pelvic hematoma	1 (0.9)	3 (3.1)	
Intestinal	1 (0.9)	1 (1)	
fever	0	2 (2.1)	
Pelvic abscess	2 (1.7)	2 (2.1)	
Vaginal dehiscence	1 (0.9)	0	
Long-term complications, grade II-IV* (%)			
No	114 (96.6)	95 (97.9)	0.556
Lymphedema	0	1 (1)	
Chronic pelvic pain	2 (1.7)	1 (1)	
Hernia	1 (0.9)	0	
Urgent urinary incontinence	1 (0.9)	0	

Grade II-IV*: according Claven-Dindo Classifications; chi-square test or Fisher's exact test for the comparison of categorical variables.

Figure S2. Learning-curve of surgical time from the introduction of robot-assisted program.

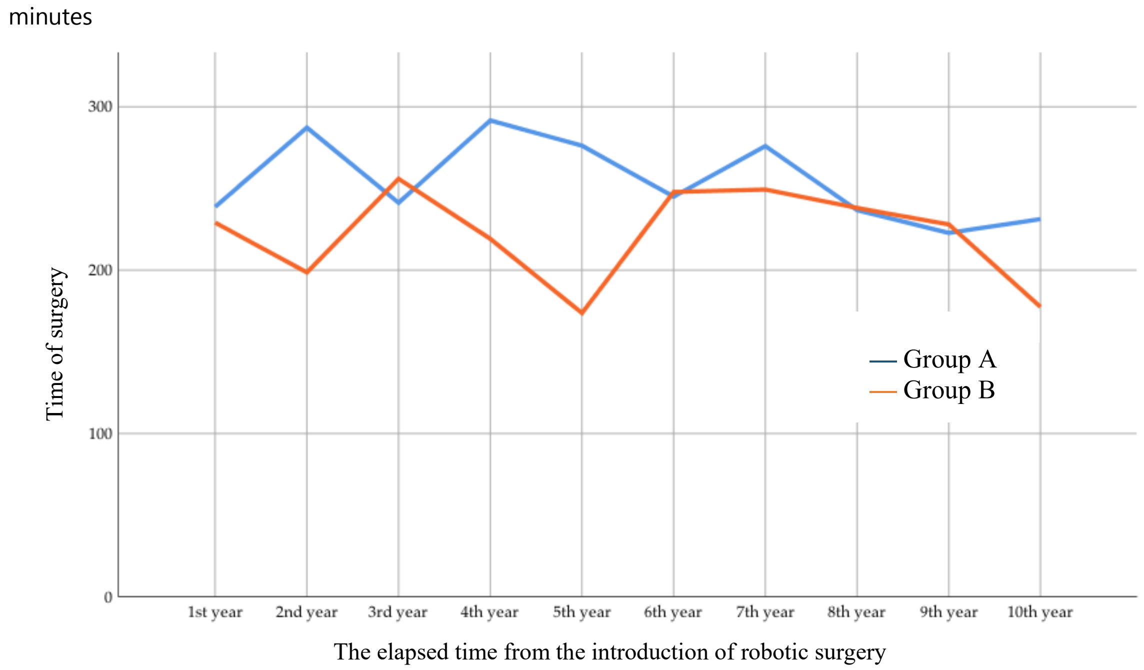

Table S2. Number of total radical hysterectomies per center.

Center\year	2009	2010	2011	2012	2013	2014	2015	2016	2017	2018
a)	11	12	11	7	11	3	7	13	5	3
b)	12	10	12	6	10	8	10	10	10	13
c)	5	4	5	5	4	5	4	6	5	8
d)	0	4	17	9	5	11	7	5	13	16
e)	5	3	8	6	6	5	5	3	2	4
